# Trend-analysis reveals real and placebo rtms effects on addiction craving: a case-control observational study

**DOI:** 10.3389/fpsyt.2024.1441815

**Published:** 2025-03-21

**Authors:** Elias Paolo Casula, Francesca Chieco, Magdalini Maria Papaioannou, Fabio Frizzarin, Lorenzo Rocchi, Arianna Camporese

**Affiliations:** ^1^ Department of System Medicine, University of Tor Vergata, Rome, Italy; ^2^ Servizio Dipendenze (SER.D.), Local Public Care Services (ULSS 6), Padua, Italy; ^3^ Department of Medical Sciences and Public Health, University of Cagliari, Cagliari, Italy

**Keywords:** addiction, craving, cocaine, gambling, rTMS, DLPFC, trend analysis, mixed models

## Abstract

**Background and aims:**

Repetitive transcranial magnetic stimulation (rTMS) can be a potential therapeutic tool for the treatment of addiction, thanks to its ability to non-invasively modulate cortical excitability. In the present study, we investigated the short- and the long-term rTMS effects on craving behaviour and psychopathological symptoms in a sample of individuals suffering from gambling and cocaine use disorder.

**Methods:**

42 individuals (age: 40.7 ± 9.5 years; 40 M) underwent an initial screening testing craving behaviour, by means of visual analogue scales, and psychopathological symptoms, by means of Symptom Checklist-90-R. Participants were subsequently assigned to a real or sham (placebo) rTMS treatment of 2 weeks delivered over the left dorso-lateral prefrontal cortex. To assess the short- and long-term effects of rTMS, participants were evaluated again after 1, 2, 4, 8, 12, 16, 20 and 24 weeks.

**Results:**

After an initial similar trend in the craving behaviour of the two groups, our trend analysis showed a long-lasting decrease (until 24 weeks) in the real-rTMS group, following a linear trend (p<0.001); whereas the sham-rTMS group progressively returned to the initial level after about 12 weeks, following a quadratic trend (p<0.001). In addition, we observed moderate-to-strong correlations (0.4<rho<1) showing that placebo effects of rTMS were stronger in individuals showing higher level of psychopathological symptoms for the first 4 weeks.

**Conclusions:**

Our results supported a long-term rTMS efficacy for cocaine and gambling craving, for which evidence was still lacking, and the correlation of short-lasting placebo effects and psychopathological symptoms.

## Introduction

In the last twenty years, the neurobiological underpinnings of addiction has been investigated in a considerable number of studies (1). Animal models show a close link between addiction and the activation of specific reward regions of the brain that regulate the release of dopamine (2, 3). Specifically, addictive substances or behaviours increase the release of dopamine causing a reward signal that, in turn, create associative learning or conditioning. When natural rewarding behaviours, such as food intake and sex, are repeated over time, the release of dopamine decreases in response to the reward; by contrast, with the intake of addictive drugs, dopamine release steadily increases. For this reason, compulsive behaviour is more common with addictive drugs intake than with natural rewards (4). Recent neuroimaging studies showed that the prefrontal cortex (PFC), a large associative area in the frontal lobe, play a key role in the development of addiction behaviour (5). It is well-known that the PFC, and in particular its dorso-lateral portion (DLPFC) represents the key hub of the executive control network and is particularly involved in decision-making, self-control and continuous monitoring of behaviour (6). In addiction all these processes are impaired. The choice of immediate reward over delayed gratification is a typical behavioural pattern of individuals suffering from craving behaviour, along with impulsivity, compulsivity and risk tendencies. Accordingly, one of the key pathophysiological findings in addiction is the widespread metabolic hypoactivity observed in the dorsal prefrontal network, and in particular in the DLPFC, resulting in impaired ability to inhibit craving (7). In this light, the choice of the DLPFC as a therapeutic target for addiction is not surprising.

Due to the lack of a clear and replicable efficacy of pharmacological and psychological treatments, a standard therapy for addiction has not been devised yet (8). Repetitive transcranial magnetic stimulation (rTMS) can be a potential therapeutic tool for the treatment of addition, thanks to its ability to non-invasively modulate cortical excitability (9). Its effectiveness has already been demonstrated in patients with depression, where the repeated application of rTMS on the DLPFC has been shown to improve mood and modulate local cortical metabolic activity (10). The mechanisms of action of rTMS are not entirely clear but they likely involve changes in the efficiency of cortical synapses. When applied at a high frequency (i.e., >1 Hz), rTMS increases cortical excitability, similar to the long-term potentiation (LTP) observed in cellular models. On the contrary rTMS reduces cortical excitability when applied at a low frequency (i.e., <=1Hz), likely by long-term depression (LTD)-like mechanisms (11).

In the field of addiction, most rTMS studies have focused on the reduction of craving, which is considered the most dysfunctional symptom in gambling disorder (GD) and cocaine use disorder (CUD) (12). Although there is no agreement on the optimal rTMS parameters for craving reduction, previous work indicate that high-frequency rTMS (≥ 5 Hz) applied over the left DLPFC produces consistent craving reduction in CUD (13–15) and GD (16). These studies showed a reduction in cocaine craving after a single rTMS session (13) or few days of treatment (14), confirmed by a reduction in the number of cocaine-free urine drug tests (15). Although promising, these findings are limited by the short-term monitoring of the effects and by the use of suboptimal parametrical statistical approaches, which are not optimized for the analysis of repeated-measures clinical designs. In the present study, we used different statistical approaches whose flexibility took into account each data variable distribution, score dependency and the individual trend followed by each participant. Our aim was to investigate the short- and the long-term effects (up to six months) of a 2-week protocol of high-frequency rTMS, delivered on the left DLPFC, in a sample of individuals suffering from GD or CUD. We adopted a sham-controlled, double-blind protocol consisting of 5 daily sessions for two weeks, with periodic evaluations of participants’ craving. Our primary outcome was self-reported craving, while we assessed psychopathological symptoms as a secondary outcome.

## Methods

### Participants criteria

Inclusion criteria for participants were i) age between 18 and 65 years, ii) a diagnosis of GD or CUD based on DSM-V criteria, ii) negative pregnancy test. Exclusion criteria were i) other neurological or severe psychiatric disorders that could interfere with the protocol, ii) the use of proconvulsant drugs. All participants gave their written informed consent before testing and did not have exclusion criteria for TMS (17). The experimental protocol was approved by the local ethics committee and was carried out in accordance with the ethical standards of the 2013 Declaration of Helsinki. The appropriateness of our sample size was established by a power calculation based on the effect size of a previous study using a similar rTMS protocol in cocaine craving (16). The power analysis was performed with G*Power software, which indicated that 30 participants would be required to detect an effect with a power of 0.95 and an alpha level of 0.05.

### Experimental procedure and clinical evaluation

Sixty-five individuals, admitted to the Public Service for Addiction (SER.D.) of Monselice (Veneto, Italy) between January 2019 and April 2021 for cocaine addiction or gambling were screened for possible inclusion in the study. Before enrolment, patients needed to be in the same drug regime for at least 3 months, this was not modified during the study protocol. Eligible participants underwent an initial screening (W0) with the following tests: i) two 10-point Visual Analog Scales (VAS) to assess craving in terms of intensity (VAS_int_) and frequency (VAS_freq_) of addiction behaviour and ii) the Symptom Checklist-90-R (SCL-90-R), a 90-item test to evaluate general psychopathological symptoms (18). In particular, we focused on three global indices of distress: Global Severity Index (SCL-90_GSI_), Positive Symptom Distress Index (SCL-90_PSDI_) and Positive Symptom Total (SCL-90_PST_), which are computed based on all the sub scales of the SCL-90-R.

Once the initial evaluation (W0) was completed, participants were assigned to a “real-rTMS” group, receiving active treatment, or to a “sham-rTMS” group, receiving a sham placebo treatment (see next paragraph for technical details). Group assignment was pseudo-randomized so that the two groups were matched for age, sex and pharmacological therapy and was performed by a technician who administered rTMS (FF) and was not divulged to other investigators. The rTMS treatment was delivered daily, from Monday to Friday, for 2 weeks. To assess the effects of the rTMS on craving, we tested VAS_int_ and VAS_freq_ at the following time points: half treatment, i.e., after one week of rTMS (W1); at the end of the treatment, i.e., after two weeks of rTMS (W2); after 4, 8, 12, 16, 20 and 24 weeks from the end of the treatment (W4-W24). To assess rTMS effects on psychopathological symptoms, we administered SCL-90-R (18) at the following time points: after 12 weeks from the treatment (W12) and after 24 weeks from the treatment (W24). Clinical evaluation was performed by experienced clinicians (AC and FC) who were blind to the patient’s group assignment.

### Repetitive transcranial magnetic stimulation protocol

rTMS was carried out using a Magstim Rapid^2^ magnetic biphasic stimulator connected with a figure-of-eight coil with a 70-mm diameter (Magstim Company, Whitland, UK) that generates a maximum magnetic field of 2.2 T. Each daily stimulation session consisted of 90 trains of 3 s, delivered at 10 Hz, with an inter-train interval of 10 s. These parameters were established based on a previous study using a shorter version of the present protocol (16) and on the most recent meta-analysis investigating the effects of rTMS in addiction (19). Based on this review, the 10-Hz frequency appear to be the most used and efficient in reducing craving beyond providing the advantage to be less discomfortable compared to other protocols delivering a higher number of pulses, e.g. 20-Hz and theta-burst stimulation, The entire session lasted approximately 20 minutes. Intensity of stimulation was based on the resting motor threshold (RMT), defined as the lowest intensity producing MEPs of >50 μV in at least five out of 10 trials in the relaxed first dorsal interosseous (FDI) muscle of the right hand (20). RMT was assessed over the optimal cortical site to elicit MEPs in the right FDI, termed “motor hotspot”, identified by positioning the coil approximately over the medial portion of the left central sulcus and moving it laterally by 0.5 cm steps. During treatment, the coil was positioned over the left DLPFC and its location was constantly monitored using the Softaxic neuronavigation system (EMS, Bologna, Italy) coupled with a Polaris Vicra infrared camera (NDI, Waterloo, Canada). The coil was positioned 5 cm anterior the motor hotspot, as previously performed in studies targeting the DLPFC (21–23). For active treatment, the coil handle was kept 45° away from the midline. For sham treatment, stimulation was applied using the same parameters but the coil was kept perpendicular to the scalp, so that no current was induced in the brain. This method, compared to other control conditions, provides the advantage to keep the same auditory and scalp sensation as in active stimulation and seems of high efficiency in persons naïve to TMS (24), like the participants in the present study. Intensity of stimulation was set at 90% of the RMT to reduce discomfort in patients. Such reduction of intensity is commonly adopted when stimulating the DLPFC (13, 25, 26) and does not reduce the efficacy of stimulation, given that this area has a lower scalp-to-cortex distance than M1 (27).

### Statistical analyses

All analyses were run with R version 3.6.1. Normal distribution of end-point variables was assessed by means of Shapiro-Wilks’ test. The level of statistical significance was set at α=0.05. Homogeneity between the means in the baseline characteristics and ongoing drug therapy (divided in five categories: antidepressants, mood stabilizers, antipsychotic, benzodiazepines and aversive drugs) between the two groups were assessed with independent t-test, Mann-Whitney test or χ^2^ depending on the type of variable (categorical or continuous) and its distribution. The longitudinal assessment of the end points across groups was performed through LMM or GLMM, depending on data distribution, for repeated measures with a random intercept to account for individual differences at baseline and for changes at follow-up points. The dependent variables for the models were: VAS_int_, VAS_freq_ as primary outcomes; SCL-90_GSI_, SCL-90_PSDI_ and SCL-90_PST_ as secondary outcomes. For the analysis of VAS, independent factors were “rTMS” (real vs. sham), “time” (W0; W1; W2; W4; W8; W12; W16; W20; W24), “group” (CUD vs. GD) and all their 2-way and 3-way interactions. For the analysis of SCL-90, we separately considered the three distress indexes and the independent factors were “rTMS” (real vs. sham), “time” (W0; W12; W24), “group” (CUD vs. GD) and all their 2-way and 3-way interactions. To test for possible effects of age and RMT we inserted these variables as covariates in all the models.

Significant effects of LMM/GLMM analyses were further evaluated with simple and polynomial contrasts. Simple contrast analysis compared the dependent variable values at the baseline level (W0) with all the subsequent follow-ups; this analysis was conducted to observe if rTMS treatments produced significant changes in variables across the full time course. Polynomial contrasts assess the goodness of fit of different trends in the dependent variable across the time points of evaluation. Specifically, we took into account three models:

(1) a linear model, to model a trend in which the dependent variable constantly changes over time, either increasing or decreasing, using the following linear function:


y=mx+b

(2) a quadratic model, to model a trend in which the dependent variable tends to flat out or raise up over time, using the quadratic function:


$$y=ax^{2}+bx+c$$

(3) a cubic model, to model a trend in which the dependent variable tends to seesaw or fluctuate up and down, using the cubic function:


$$y=ax^{3}+bx^{2}+cx+d$$

Finally, we were interested to assess linear relationships between the initial psychopathological symptomatology, i.e., SCL-90_GSI_, SCL-90_PSDI_ and SCL-90_PST_ scores at the initial evaluation (W0), and the efficacy of rTMS on craving, i.e., the VAS_int_ and VAS_frq_ score changes between the initial evaluation (W0) and the following time points. This analysis was conducted separately for the two groups (real, sham) by computing the Pearson’s or the Spearman’s correlation coefficient, depending on data distribution.

## Results

### Baseline characteristics of the participants

Of the sixty-five participants screened, forty-five satisfied the inclusion criteria and were recruited in the study. Three participants dropped out after the first evaluation (two patients voluntarily dropped out, one patient found rTMS excessively uncomfortable to complete the protocol) and were excluded from the analyses. Thus, a total of forty-two participants (nineteen in the real-rTMS group and twenty-three in the sham-rTMS group) were included, of these, 22 participants suffered from CUD and 20 from GD. **Table 1** summarizes the baseline characteristics of the two groups. No differences were observed in demographic characteristics in terms of age (real: 40.95 ± 9.87 years; sham: 40.43 ± 9.38 years; t(40)=0.172; p=0.864; d=0.053), sex (real: 18 male, 1 female; sham: 22 male, 1 female; χ^2^ = 0.0192; p=0.890; phi=0.021). No differences were observable in the drug therapy of the two groups for antidepressants (real: 8 (42.1%) participants; sham: 8 (34.8%) participants; U(40)=-0.250; p=0.803), mood stabilizers (real: 3 (15.8%); sham: 3 (13%); U(40)=-0.297; p=0.766), antipsychotics (real: 4 (21.1%); sham: 4(17.4%); U(40)=-0.137; p=0.891), benzodiazepines (real: 3 (15.8%); sham: 4 (17.4%); U(40)=-0.199; p=0.842), aversive drugs (real: 2 (10.5%); sham: 1 (8.7%); U(40)=-0.765; p=0.445). No differences were observable in the initial evaluation measures, including RMT (real: 55.63% ± 10.16% MSO; sham: 56.86% ± 7.27% MSO; t(40)=-0.459; p=0.648; d=0.142), VAS_int_ (real: 6.73 ± 2.62; sham: 5.91 ± 3.31; t(40)=-0.867; p=0.391; d=-0.269), VAS_freq_ (real: 4.94 ± 3.25; sham: 5.00 ± 3.35; t(40)=0.061; p=0.951; d=0.019); SCL-90_PST_ (real-rTMS: 51.58 ± 16.55; sham-rTMS: 48.36 ± 16.96; t(40)=0.612; p=0.544; d=0.191), SCL-90_PSDI_ (real: 1.94 ± 0.67; sham: 1.73 ± 0.42; t(40)=-1.241; p=0.222; d=-0.384) and SCL-90_GSI_ (real: 1.14 ± 0.66; sham: 0.92 ± 0.41; t(40)=-1.318; p=0.195; d=-0.408).

**Table 1:** Baseline demographic, drug therapy and clinical characteristics (mean ± SD) of the real-rTMS and sham-rTMS groups with p-values testing for significant difference between the two groups.

	Real-rTMS group	Sham-rTMS group	Group difference
Sex, male (f)	18 (1)	22 (1)	p=0.891
Age, years	40.95 ± 9.87	40.43 ± 9.39	p=0.862
RMT, %	55.63 ± 10.16	56.87 ± 7.27	p=0.651
Gambling disorder	7 (36.8%)	8 (34.8%)	p=0.891
Cocaine use disorder	12 (63.2%)	15 (65.2%)	p=0.891
Antidepressants	8 (42.1%)	8 (34.8%)	p=0.803
Mood stabilizers	3 (15.8%)	3 (13%)	p=0.766
Antipsychotics	4 (21.1%)	4 (17.4%)	p=0.891
Benzodiazepines	3 (15.8%)	4 (17.4%)	p=0.842
Aversive drugs	2 (10.5%)	1 (8.7%)	p=0.445
Gambling disorder	7 (36.8%)	8 (34.8%)	p=0.891
SCL-90 GSI	1.14 ± 0.66	0.921 ± 0.41	p=0.321
SCL-90 PSDI	1.94 ± 0.67	1.73 ± 0.42	p=0.372
SCL-90 PST	51.58 ± 16.55	48.36 ± 16.97	p=0.542
VAS intensity	6.73 ± 2.63	5.91 ± 3.31	p=0.591
Vas frequency	4.94 ± 3.25	5.00 ± 3.36	p=0.962

### Primary outcome measure: addiction craving

To assess the effects on VAS_int_ we fitted a LMM, whereas the effects on VAS_freq_ were tested with a GLMM with negative binomial distribution and log-link function (based on the Akaike criteria), since the residual distribution was not normal (Shapiro-Wilk test p<0.05). The general equation of the model was:


(1) $$\begin{array}{c} VAS∼1+rTMS+time+group+age+RMT+rTMS:time\\ +group:rTMS+group:time+group:rTMS:time\\ +(1|patient) \end{array}$$

**Figure 1:**
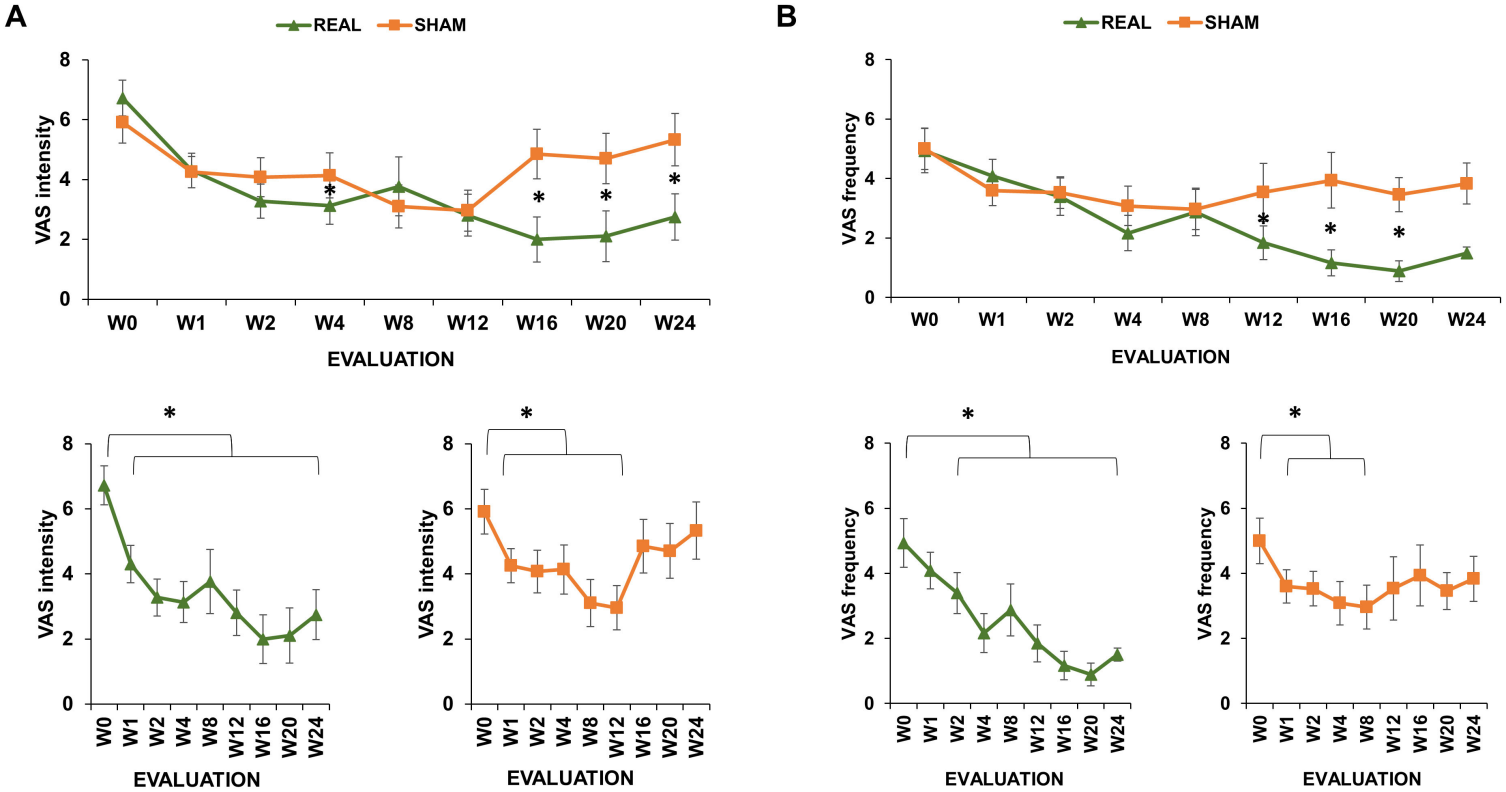
Visual Analogue Scale (VAS) scores of real-rTMS and sham-rTMS group. VAS scores measuring craving intensity **(A)** and frequency **(B)** at the initial evaluation (W0), after 1 week of treatment (W1), after 2 weeks of treatment (2W) and the subsequent follow-ups: after 4 (W4), 8 (W8), 12 (W12), 16 (W16), 20 (W20) and 24 weeks (W24) from the end of the treatment. Green line depicts the trend of the group receiving real-rTMS; orange line depicts the trend of the group receiving sham-rTMS. Error bars depict the standard error of the mean. * indicates p<0.05.

The analysis of VAS_int_ revealed a significant rTMS*time interaction [F(8,197.4)=2.404; p=0.017] (**Figure 1A**). No other significant main effects or interactions were observed (all p values > 0.05). Simple contrast analysis of the rTMS*time interaction showed a significant reduction of the initial (W0) VAS_int_ in the real group, compared to the sham group, at the following time points: W4 (β=-2.014; CI=[-3.920;-0.108]; p=0.04), W16 (β=-3.241; CI=[-5.498;-0.983]; p=0.005), W20 (β=-2.614; CI=[-4.842;-0.387]; p=0.022) and W24 (β=-3.434; CI=[-5.611;-1.256]; p=0.002). By contrast, we did not observe significant differences between the two groups at W1 (β=-0.441; CI=[-2.351; 1.470]; p=0.652), W2 (β=-1.733; CI=[-3.624; 0.156]; p=0.074); W8 (β=-0.467; CI=[-2.604; 1.669]; p=0.669) and W12 (β=-0.978; CI=[-3.061; 1.104]; p=0.358) (**Figure 1A**). Within-group comparisons conducted with simple contrasts showed a significant reduction of the initial (W0) VAS_int_ at all the time points (all ps<0.001) in the real group, whereas in the sham group this effect was significant for W1 (p=0.002), W2 (p=0.009), W4 (p=0.017), W8 (p<0.001) and W12 (p<0.001), but not for W16 (p=0.104), W20 (p=0.051) and W24 (p=0.167) (**Figure 1A**). Polynomial contrasts showed that VAS_int_ level throughout the different time points was accurately described by a linear model (β=-3.222; p<0.001) in the real-rTMS group, and by a quadratic model in the sham-rTMS group (β=2.071; p<0.001).

The analysis of VAS_frq_ revealed a significant rTMS*time interaction [χ^2^(8)=27.554; p<0.001] (**Figure 1B**). No other significant main effects or interactions were observed (all ps>0.05). Simple contrast analysis of rTMS*time interaction showed a significant reduction of the initial (W0) VAS_frq_ in the real group, compared to the sham group, at W12 (exp(B)=0.551; CI=[0.310; 0.981]; p=0.025), W16 (exp(B)=0.340; CI=[0.157; 0.737]; p=0.006) and W20 (exp(B)=0.367; CI=[0.171; 0.788]; p=0.008), whereas we did not observe significant differences between the two groups at W1 (exp(B)=1.488; CI=[0.925; 2.394]; p=0.101), W2 (exp(B)=1.121; CI=[0.706; 1.780]; p=0.628), W4 (exp(B)=0.723; CI=[0.441; 1.185 p=0.198), W8 (exp(B)=1.239; CI=[0.616; 2.490]; p=0.548) and W24 (exp(B)=0.639; CI=[0.366; 1.115]; p=0.115). Within-group analyses conducted with simple contrasts showed a significant reduction of the initial (W0) VAS_frq_ in all the time points (all ps<0.001), except for W1 (p=0.219), in the real group, whereas in the sham group this effect was significant at W1 (p=002), W2 (p=0.009), W4 (p=0.011) and W8 (p=0.001), but not at W12 (p=0.081), W16 (p=0.204), W20 (0.056) and W24 (p=0.063) (**Figure 1B**). Polynomial contrasts showed that VAS_frq_ level throughout the different time points was accurately described by a linear model (β=-1.147; p<0.001) in the real group, and by a quadratic model in the sham group (β=0.376; p=0.017).

### Secondary outcome measure: psychopathological symptomatology

Our secondary outcomes (SCL-90_GSI_, SCL-90_PSDI_ and SCL-90_PST_) were analysed by fitting the following GLMM with negative binomial distribution with log-link function (based on the Akaike criteria), since the residual distribution was not normal (Shapiro-Wilk test p<0.01) (see general eq. 2):


(2) $$\begin{array}{c} test∼1+rTMS+time+group+age+RMT+rTMS:time\\ +group:rTMS+group:time+group:rTMS:time\\ +(1|patient) \end{array}$$

**Figure 2:**
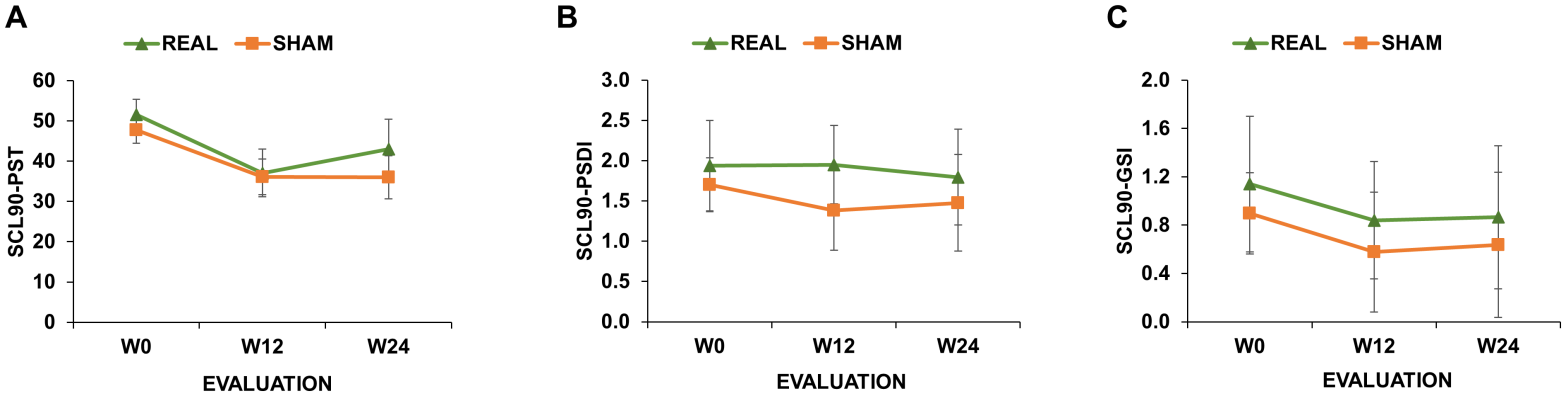
Symptom Checklist 90-R (SCL-90) scores of real-rTMS and sham-rTMS group. SCL-90 scores measuring Positive Symptom Total [SCL-90_PST_; **(A)**]; Positive Symptom Distress Index [SCL-90_PSDI_; **(B)**] and Global Severity Index [SCL-90_GSI_; **(C)**] at the initial evaluation (W0), after 12 (W12) and 24 weeks (W24) from the end of the treatment. Green line depicts the trend of the group receiving real-rTMS; orange line depicts the trend of the group receiving sham-rTMS. Error bars depict the standard error of the mean.

These analyses did not reveal any significant main effect or interactions (all ps>0.05; **Figure 2**).

### Correlations between psychopathological symptomatology and craving

**Figure 3:**
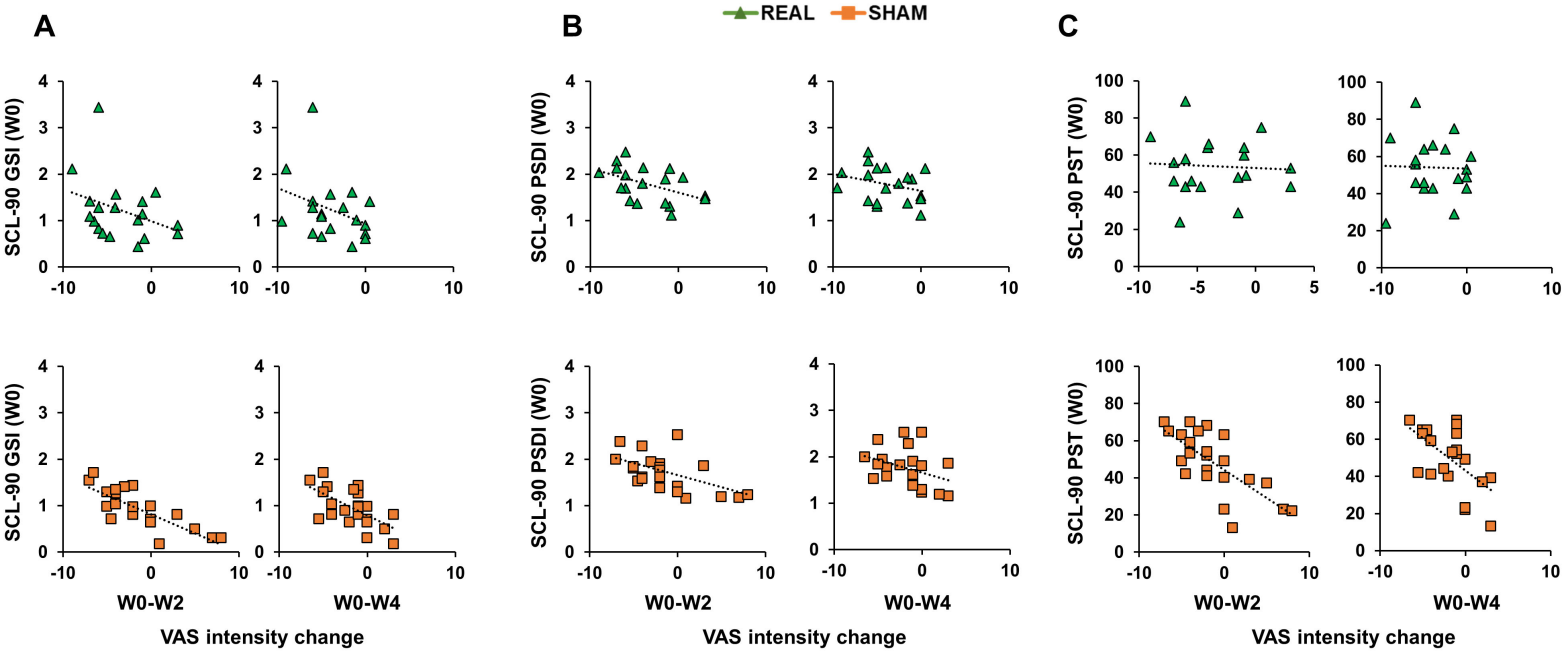
Correlations between Symptom Checklist 90-R (SCL-90) initial scores and Visual Analogue Scale (VAS) intensity change. Scatterplots of the correlations between SCL-90 scores measuring Positive Symptom Total [SCL-90_PST_; **(A)**]; Positive Symptom Distress Index [SCL-90_PSDI_; **(B)**]; Global Severity Index [SCL-90_GSI_; **(C)**] and the change in VAS intensity from the initial evaluation to the end of the treatment (W0-W2) and to the follow-up after 4 weeks from the treatment (W0-W4). Green triangles depict the cases receiving real-rTMS; orange squares depict the cases receiving sham-rTMS.

**Figure 4:**
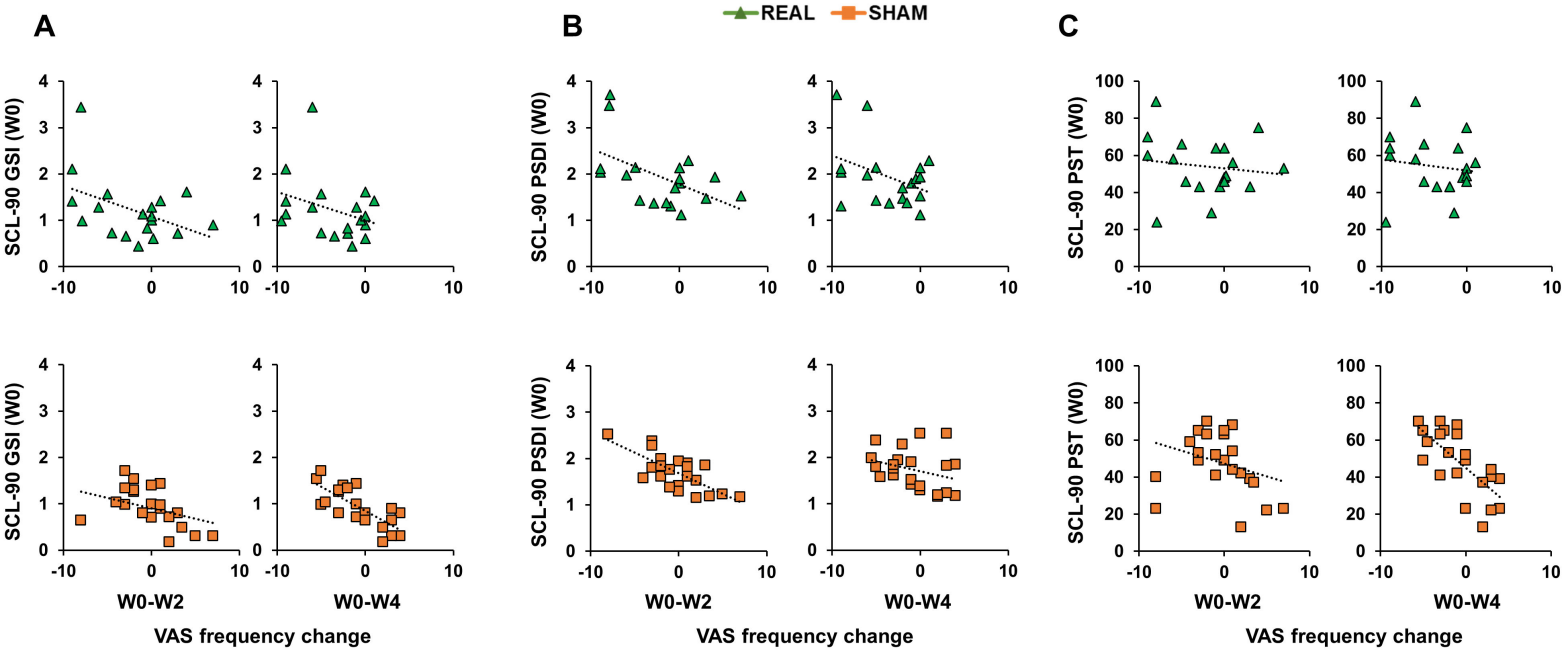
Correlations between Symptom Checklist 90-R (SCL-90) initial scores and Visual Analogue Scale (VAS) frequency change. Scatterplots of the correlations between SCL-90 scores measuring Positive Symptom Total [SCL-90_PST_; **(A)**]; Positive Symptom Distress Index [SCL-90_PSDI_; **(B)**]; Global Severity Index [SCL-90_GSI_; **(C)**] and the change in VAS frequency from the initial evaluation to the end of the treatment (W0-W2) and to the follow-up after 4 weeks from the treatment (W0-W4). Green triangles depict the cases receiving real-rTMS; orange squares depict the cases receiving sham-rTMS.

Correlation analyses were conducted using Spearman’s coefficient given that all SCL-90 scores distributions were not normal (Shapiro-Wilk test <0.01). When considering the real group, analyses did not reveal any significant correlation (all p values > 0.05; **Figures 3**, **4**). When considering the sham group, analysis revealed a significant negative correlation between all three SCL-90 scores and the VAS score changes at W2, considering both VAS_int_ (SCL-90_GSI_ – VAS_int_: rho=-0.795, p<0.001; SCL-90_PSDI_ – VAS_int_: rho=-0.481, p=0.020; SCL-90_PST_ – VAS_int_: rho=-0.709, p<0.001; **Figure 3**) and VAS_frq_ (SCL-90_GSI_ – VAS_frq_: rho=-0.445, p=0.033; SCL-90_PSDI_ – VAS_int_: rho=-0.652, p<0.001; SCL-90_PST_ – VAS_int_: rho=-0.486, p=0.022; **Figure 4**). Correlations were also significant when considering the VAS score changes at W4, both for VAS_int_ (SCL-90_GSI_ – VAS_int_: rho=-0.727, p<0.001; SCL-90_PSDI_ – VAS_int_: rho=-0.417, p=0.048; SCL-90_PST_ – VAS_int_: rho=-0.647, p=0.001; **Figure 4**) and VAS_frq_ (SCL-90_GSI_ – VAS_frq_: rho=-0.467, p=0.025; SCL-90_PSDI_ – VAS_int_: rho=-0.420, p=0.046; SCL-90_PST_ – VAS_int_: rho=-0.558, p=0.007; **Figure 4**).

## Discussion

The main aim of the present study was to investigate the short- and long-term effects of a two-week protocol of high-frequency rTMS on craving in CUD and GD. Craving was assessed in terms of intensity and frequency with VAS scores for 6 months, i.e. 24 weeks, after the treatment. The results of our work showed a similar initial trend in craving behaviour after both real and sham rTMS. However, starting from the twelfth week of observation, the groups diverged: in the sham-rTMS group craving frequency (from W12) and intensity (from W16) progressively returned to the initial level, whereas the same variables showed a longer lasting decrease (until W24) in the real-rTMS group. Such difference was confirmed by our trend analysis, which is based on the application of polynomial contrasts and is sensible in detecting different trends in repeated-measures datasets. In the group treated with real rTMS, the VAS scores progressively decreased from the initial evaluation (W0) to the last follow-up point (W24), following a negative linear trend. Differently, in the sham group, VAS scores showed an initial decrease (until W8-W12) followed by a raise in the latest follow-up points, compatible with a quadratic trend. Notably, these results were shared by participants suffering CUD and GD, as suggested by the lack of significance of factor “group” in our analyses. This result support the notion that GD and CUD share common neurobiological dysfunction and behavioural patterns (28–30). Accordingly, GD is currently included in the diagnostic category of substance-related and addictive disorders based on the Diagnostic and Statistical Manual of Mental Disorders fifth edition [DSM-5; Regier, Kuhl, e Kupfer 2013 (31)]. But what is the physiological interpretation of the rTMS effects on craving when applied to the left DLPFC?

As mentioned in the introduction, high-frequency rTMS is known to promote synaptic plasticity based LTP-like mechanisms by acting on cortico-striatal axons (11). In the context of addiction, the efficacy of TMS may be due to transient increase in dopamine levels in the limbic areas interconnected with the DLPFC (32–34). In particular, it has been hypothesized that rTMS promotes dopamine secretion in mesolimbic and mesostriatal pathways through the DLPFC projections to the ventral tegmental area, thus resulting in a restoring of dopaminergic dysfunction (19, 34–36).

A noteworthy result of the present study is the initial craving reduction in the group treated with sham rTMS. This observation can be interpreted in two different ways. First, it is conceivable that the common trend of the two groups in the first 8 weeks is due to the pharmacological therapy that both groups followed as patients of the SER.D. In this light, the longer and stronger craving reduction observed in the real-rTMS group, can be interpreted as a “boost” of the effects of the ongoing pharmacological therapy caused by rTMS of the DLPFC. This is what we observed in our trend analysis: from the third month after the treatment (W12) the sham-rTMS group showed a re-increase of craving to the initial levels before the treatment (W0), whereas the craving levels of the real rTMS group were still significantly reduced after six months (W24). This is in agreement with several studies that applied rTMS as an “add-on” therapy to boost the effects of drugs (37), psychotherapy (38) and physical therapy (39). In a different perspective, the initial craving reduction in participants treated with sham stimulation group can be interpreted as a placebo effect. This should not be surprising given the well-known placebo effects related to brain stimulation techniques (40). In the field of addiction, rTMS placebo effects have been reported (for a review see Amerio et al., 2023), although their influence have not been discussed in depth as in depression (41), obsessive-compulsive disorder (42) and motor rehabilitation (43). This is relevant since individuals suffering from GD and CUD often presents several psychopathological symptoms [e.g. depressive disorder, enhanced stress, anxiety; Martin et al., 1977 (44)] that are strictly related to the susceptibility to placebo effects (45). Indeed, a large piece of evidence showed that personal beliefs and expectations can strongly affect the response to a therapy and, in particular, moderate-to-high levels of psychopathology are associated to the magnitude of placebo effects (46). This notion is in strict agreement to the correlation analysis of our study. Here we found moderate-to-strong correlations (0.4<rho<1) between the efficacy of rTMS on craving (i.e. measured with VAS score change from the W0 initial evaluation) and the presence of psychopathological symptoms (SCL-90 scores) in the sham-rTMS group, but not in the real-rTMS group. Importantly, these correlations were significant only considering the first two post-rTMS evaluations, i.e. W2 and W4, and not the later follow-ups (W8-W24). Based on the above considerations, the interpretation of this result is quite straightforward: participants showing the highest level of psychopathological symptoms were also the ones who perceived the strongest placebo effect of sham-rTMS. However, these effects were limited to the first two follow-ups, during which the placebo effects were stronger.

A third result of the present work is the absence of rTMS effects on the level of psychopathological symptoms, as measured with SCL-90. This result is in agreement with a previous study using a similar protocol over the left DLPFC that showed an effect on craving reduction but not on SCL-90 scores (15). A number of reasons can account for this null result. First, our protocol was aimed at reducing craving behaviour, which was also the primary outcome of the study, thus it can be conceivable that the rTMS parameters were not optimal to produce an effect on psychopathological symptoms. Second, rTMS effects over SCL-90 scores were assessed only in two distant follow-up points, i.e., W12 and W24, so that we did not have an accurate temporal resolution of the temporal trend followed by the participants, as for the VAS scores.

Our work presents some limitations. First, our sample size was mainly composed by male individuals. This is a common bias for studies in addiction given the higher prevalence of addiction in males [for a review see (36)]. Thus, any conclusion of the present study, similar to other rTMS studies in the same field, should be restricted to male individuals. Second, we were not able to collect urine drug tests in our participants. Third, we did not have individualized MRI for every participant; therefore, it was not possible to localize the left DLPFC based on individual anatomy in all subjects. To minimize errors due to individual variability, we localized the DLPFC hotspot based on the individual M1 hotspot, which was functionally defined as the spot producing highest MEPs, measured with EMG. Finally, the effect monitoring of the present study is limited to six months after the treatment; thus, no conclusion can be inferred after this time point. Thus, it can be conceivable that the effects of our protocol are limited to this time range. In this regard, future studies need to perform a longer monitoring of the effects and consider to add a “maintenance phase” of the protocol in which weekly or bi-weekly rTMS session are administered to the patient to “maintain” neuromodulatory effects (47).

In conclusion, the present study demonstrates long-term efficacy of a 2-week high-frequency rTMS protocol on craving from CUD and GD. Our main results showed that rTMS produced a sustained reduction of craving intensity and frequency until 6 months from the end of the treatment. Interestingly, we also observed an initial craving reduction even in participants following a sham-rTMS protocol, likely due to a placebo effect. The main contributions of the present results are detailed below. First, we demonstrated a long-term rTMS efficacy not only for cocaine craving, but also for gambling disorder, for which evidence was still lacking. This result supports the use of the rTMS as a treatment for general addiction and its long-term efficacy, although future studies need to assess its efficacy in other kinds of addiction and in longer time windows. Second, we carefully adopted an optimized statistical approach for the evaluation of our data. In detail, we carefully assessed data distribution of each single dependent variable and applied an *ad-hoc* statistical model showing the best fit for each dataset. To this aim, we chose the statistical test distribution and the link function of our generalized linear mixed models based on their goodness of fit, as computed with the Akaike information criterion, an estimator of prediction error in statistical models. Each patient was inserted as a level of a cluster variable so that the model’s intercept could vary depending on the individual clinical evolution. Finally, we adopted polynomial contrasts to perform a trend analysis able to fit a linear or non-linear function to the different temporal trends followed by the patients. Although fundamental, these aspects are often ignored in clinical trials using standard parametrical model that does not take into account important information of the data, such as residual distribution and individual trends.

## Data Availability

The raw data supporting the conclusions of this article will be made available by the authors, without undue reservation.
